# A rocky road but worth the drive: A longitudinal qualitative study of patient innovators and researchers cocreating research

**DOI:** 10.1111/hex.13790

**Published:** 2023-06-09

**Authors:** Carolina Wannheden, Sara Riggare, Jamie L. Luckhaus, Hanna Jansson, My Sjunnestrand, Terese Stenfors, Carl Savage, Maria Reinius, Henna Hasson

**Affiliations:** ^1^ Department of Learning, Informatics, Management and Ethics Medical Management Centre, Karolinska Institutet Stockholm Sweden; ^2^ Department of Women's and Children's Health, Participatory eHealth and Health Data Uppsala University Uppsala Sweden; ^3^ Department of Learning, Informatics, Management and Ethics Division of Learning, Karolinska Institutet Stockholm Sweden; ^4^ School of Health and Welfare Halmstad University Halmstad Sweden; ^5^ Unit for Implementation and Evaluation Center for Epidemiology and Community Medicine (CES) Stockholm Sweden

**Keywords:** cocreation, coproduction, partnership research, patient and public involvement, patient author, patient‐driven innovation

## Abstract

**Background:**

Partnership research practices involving various stakeholder groups are gaining ground. Yet, the research community is still exploring how to effectively coproduce research together. This study describes (a) key programme developments in the creation of a 6‐year partnership research programme in Sweden, and (b) explores the hopes, expectations, and experiences of patient innovators (i.e., individuals with lived experience as patients or caregivers who drive health innovations) and researchers involved in the programme during the first years.

**Methods:**

We conducted a prospective longitudinal qualitative study spanning the first 2 years of the programme. Data consisted of meeting protocols and interviews with 14 researchers and 6 patient innovators; 39 interviews were carried out in three evenly‐spaced rounds. We identified significant events and discussion themes in the meeting protocols and analyzed the interviews using thematic analysis, applying a cross‐sectional recurrent approach to track changes over time.

**Findings:**

Meeting protocols revealed how several partnership practices (e.g., programme management team, task forces, role description document) were cocreated, supporting the sharing of power and responsibilities among programme members. Based on the analysis of interviews, we created three themes: (1) *paving the path to a better tomorrow*, reflecting programme members' high expectations; (2) *going on a road trip together*, reflecting experiences of finding new roles and learning how to cocreate; (3) *finding the tempo: from talking to doing*, reflecting experiences of managing challenges and becoming productive as a team.

**Conclusions:**

Our findings suggest that sharing, respecting, and acknowledging each other's experiences and concerns helps build mutual trust and shape partnership practices. High expectations beyond research productivity suggest that we need to consider outcomes at different levels, from the individual to society, when evaluating the impact of partnership research.

**Patient or Public Contribution:**

The research team included members with formal experiences as researchers and members with lived experiences of being a patient or informal caregiver. One patient innovator coauthored this paper and contributed to all aspects of the research, including the design of the study; production of data (as interviewee); interpretation of findings; and drafting the manuscript.

## 1 INTRODUCTION

Patient and public involvement (PPI) in designing healthcare services and research is gaining ground.^1^, ^2^, ^3^, ^4^ Commonly mentioned benefits are that research becomes more relevant, acceptable, and appropriate for patients. Furthermore, the involvement of patients in setting research agendas may contribute to reducing avoidable waste in the production and reporting of research evidence.^5^ Models and frameworks have been developed for supporting PPI approaches in diverse activities, such as research agenda setting,^6^ clinical trials,^7^ drug development,^8^ as well as the production and reporting of research.^9^ In addition, guidance is available from organizations such as the National Institute for Health Research^10^ in the United Kingdom and the Patient‐Centred Outcomes Research Institute^11^ in the United States. These guidelines primarily concern one‐directional involvement strategies where researchers involve patients and public contributors. Adapting general guidance to specific research situations can be challenging.^12^

Challenges have been reported in studies where patients had no clear role or were only sporadically involved.^13^ Sporadic involvement for the purpose of informing or consulting may be interpreted as tokenism,^14^ and can be experienced as ‘virtue signalling’.^15^ It has been suggested that poor empirical evidence about the impact of PPI in research leads to lacking consensus about what constitutes effective involvement processes.^16^ A review of the literature identified 65 frameworks for PPI in research^17^; one factor highlighted as important was encouraging involvement in all research phases, starting from the project formation phase. Furthermore, creating a culture of continuous involvement and developing relationships and trust were emphasized together with building understanding about the local context and offering opportunities for training and capacity building. Challenges that were identified include the use of different terminology, lack of knowledge of specific research issues, and health‐related challenges in life that set boundaries for patients' research involvement.^17^ How these factors (or other factors) impact creating long‐term research partnerships is not yet known.

Moving toward long‐term research partnerships requires a fundamental paradigm shift in health and social care services research with members of the public collaboratively involved throughout the research process, from study design to publication.^18^, ^19^ There is strong consensus among experts that working in equal partnerships is essential.^20^ However, how to create equal partnerships is not clear from the current literature. Hickey et al.^21^ used the term coproduction of research and defined it as when ‘researchers, practitioners and the public together share power and responsibility for the work throughout’ (p. 29). The concept of coproduction in research, and related concepts, such as *cocreation*,^22^ or *partnership*,^23^ goes beyond *involving, engaging*, or *empowering* patients and public stakeholders.^24^ Coproduction requires a redistribution of power among stakeholders, moving from short‐term contacts towards more long‐term collaborations with patients and public representatives.^25^ Equitable partnerships can be formed by specifically addressing power inequalities between stakeholders.^26^ To emphasize the concept of collaboration on equitable terms without assuming that one stakeholder group involves another, we will in this paper use the terms partnership research and coproduction of research, rather than PPI.

The research community is still exploring different ways of coproducing research together with patients. The meaning of coproduction, as well as its costs and benefits, are debated among scholars in the field.^27^, ^28^ Experiences from partnership programmes are therefore highly needed,^29^ particularly those studies that take into account the perspectives and experiences of both patients and researchers involved in partnership research, which have been scarce.^30^ Given the facilitating factors that have been identified, as well as the mind shifts that are often required as one moves towards partnership, we decided to explore this process in the context of a research programme titled ‘Patients in the driver's seat’. This study aims to (a) describe key programme developments in the creation of this 6‐year (2019–2024) partnership research programme, and (b) explore the hopes, expectations, and experiences of the programme members (i.e., patient innovators and researchers) during the first programme years.

## 2 METHODS

### 2.1 Design

This study was designed as a prospective longitudinal qualitative study building on meeting protocols and interviews, collected during a period of 2 years.

### 2.2 Setting

The ‘Patients in the driver's seat’ research programme studies how five innovations developed and driven by patients or informal caregivers to support self‐care or cocare (i.e., an approach that emphasizes the combination of the patient's and healthcare's resources)^31^ are implemented in clinical practice and the daily lives of patients and their families. The innovations initially included in the programme were Genia,^32^, ^33^ Dream Catcher,^34^ CareMaps,^35^, ^36^ Patient Recovery Education,^37^ and Patient Lead Users.^38^ Each innovation was represented by individuals with lived experiences as patients or caregivers (here called patient innovators). Patient innovators participated already in the conceptualization of the research plan and two of them were coapplicants. The programme ambition as stated in the funding application was to ensure that patient innovators would have an opportunity to be engaged throughout the research process together with other programme members. A collaborative approach was intentionally adopted to enable members to have influence over the decisions made. The goal was that all members keep their expert roles but are equitable partners in the programme. Thus, for simplicity, we will subsequently use the labels patient innovator and researcher to emphasize programme members' expert roles, although we acknowledge that programme members could identify with multiple roles.

### 2.3 Data collection

Meeting protocols (*n* = 53) were collected from the initiation of the programme (January 2019) to the end of the second year (December 2020). Thirty‐nine semi‐structured interviews with programme members were carried out in three rounds: 12 interviews after programme initiation (February–March 2019), 14 at the end of the first year (November–December 2019), and 13 at the end of the second year (October of 2020). All programme members (i.e., researchers and patient innovators) were invited to participate in each interview round, regardless of position (e.g., junior, senior, short‐ or long‐term contract) or working hours spent in the programme. As the programme members were not constant throughout the study period, the respondents could vary between rounds. In total, 14 researchers and 6 patient innovators participated: five participated in all three rounds, nine participated in two rounds, and six participated in one round. Two female research assistants who were external to the programme and had training and experience in qualitative interviewing performed the interviews. The research assistants developed the interview guides with assistance from a senior researcher also external to the programme. The topic areas of the interviews were the same in all rounds (i.e., individual learning and development; roles; meaning, facilitators and barriers of cocreation; and hopes and expectations). The first two rounds were conducted in‐person or via phone if a respondent preferred; the third round was conducted via a videoconferencing system, due to the COVID‐19 pandemic. The interviews lasted 15–90 min (average 47 min), were audio‐recorded, and transcribed by an external transcription service. Member‐checking was conducted before analysis, in which participants were given the option to read their own transcripts and remove any information that they did not want to be included.

### 2.4 Data analysis

A longitudinal, inductive approach was used to thematically analyze meeting protocols and interview transcripts. Meeting protocols provided insight into key developments and discussions in the programme, and interviews provided insight into programme members' hopes, expectations, and experiences. The two data sources were analyzed and reported separately. Two researchers (M. S. and H. J.) read through all the meeting protocols, annotated them with metadata (date and type of meeting), and identified meaning units reflecting significant events and discussions. The meaning units were condensed and labeled with descriptive codes that were grouped into themes. To explore development over time, we plotted the meeting frequency and key discussion themes on a timeline. The interview data were analyzed using inductive reflexive thematic analysis to develop themes based on patterns identified in the data.^39^ We used a recurrent cross‐sectional approach, in which each interview round was coded as a separate unit, and later analyzed as a whole—comparing and combining themes.^40^ J. L. L. performed the systematic coding: she familiarized herself with the data; generated initial codes which she transferred to a virtual whiteboard in the Miro online visual collaboration platform^41^; and then sorted the codes into potential themes, separately for each interview round. After the systematic coding, a respondent patient innovator (S. R.) and researcher (C. W.) were invited to read the anonymized codes and discuss the preliminary themes with J. L. L.; this was done in several iterations. This process contributed to maintaining reflexivity as different perspectives were combined.^42^ In the final stage, J. L. L. selected illustrative quotations for the presentation of findings; these were translated from Swedish to English with minor adjustments to enhance readability. Quotations are identified by the participant number and their expert role (I = patient innovator; R = researcher).

### 2.5 Ethical considerations

We acknowledge that our study may raise some ethical concerns as all authors, except J. L. L., were actively engaged in the research programme and participated as interview respondents. J. L. L., who had no previous collaboration with any of the research programme members, was engaged in the study to analyze interviews. To protect confidentiality and mitigate bias in the analysis, J. L. L. was the only one who had access to interview transcripts and removed personal identifiers before sharing abstracted data with coauthors. Ethical approval for the study was obtained from the Swedish Ethical Review Authority (reg nr. 2019‐03849). Participants were informed that their participation was optional and were made aware of who would have access to their data and how it would be used. Written and verbal informed consent was obtained before each interview.

## 3 FINDINGS

### 3.1 Key developments in the programme

**Figure 1:**
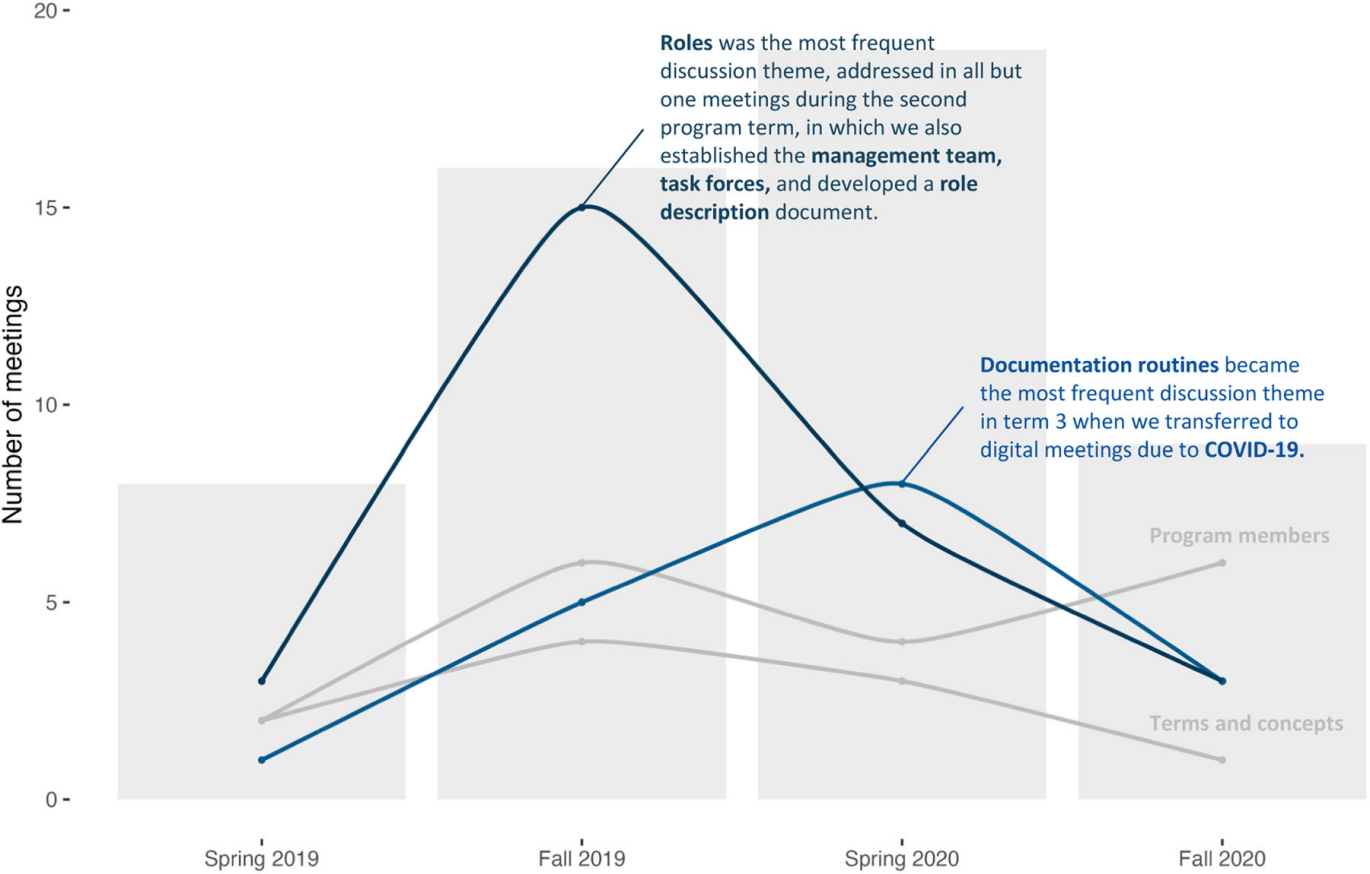
Frequency of meetings addressing each of four main discussion themes (roles, documentation routines, programme members, and terms and concepts) over time. The grey bars illustrate the total number of analyzed meetings per half‐year.

The programme started with a meeting with all involved actors in December 2018, followed by monthly meetings during the first 6 months. The project group expanded with the addition of two postdoctoral researchers recruited in autumn of 2019, whereafter the meeting intensity doubled. Recurrent discussion themes in these meetings were the programme member constellation, roles of patient innovators and researchers, documentation routines, and definitions of key concepts (Figure 1). The discussions on roles led to the formation of a project management team with equal role representation (5 + 5). The management team held monthly meetings focused on strategic issues. The first was to define programme member roles and responsibilities. A four‐person task force with equal representation was formed to propose a role description and delegation system. The resulting role description document became a guiding foundation for the programme and was regularly revisited and revised. A major revision in the role description document consisted of the addition of core programme values and principles that had been specified in a meeting with all programme members. In addition to the management team meetings, regular research meetings were held triweekly to discuss and coordinate planned and ongoing research activities, as well as actively conduct research together. All members were invited and could also bring additional stakeholders (e.g., healthcare professionals) to these meetings. Toward the end of the first year, we invited patient researcher Dana Lewis who facilitated a ‘baggage claim’ exercise.^43^ This was a team‐building exercise in which all programme members shared their prior frustrating experiences of research collaborations (i.e., baggage). By checking this baggage, the idea was to leave frustrating experiences behind and focus more on what we wanted to achieve ahead. In March 2020 (i.e., during the second year), the COVID‐19 pandemic erupted in Sweden.^44^ We smoothly shifted our meetings to a digital format, which made participation more accessible as there was no need to commute between different locations. As a result of the transition to fully digital collaboration, documentation routines temporarily became a more frequent discussion topic. Some research activities were paused, resulting in reduced meeting intensity during the second half of 2020.

### 3.2 Programme members' expectations and experiences

**Figure 2:**
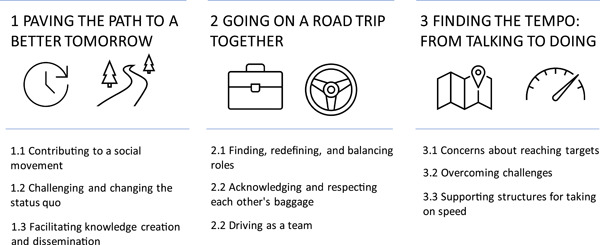
Themes and subthemes of hopes, expectations, and experiences of partnership research.

The analysis of interviews resulted in three themes, each composed of three subthemes (Figure 2).

#### 3.2.1 Paving the path to a better tomorrow

##### Contributing to a social movement

Members perceived that the programme contributed to a social movement in which patient‐driven innovations as well as partnership research are receiving increasing attention. The use of words like ‘power’, ‘source of inspiration’, and ‘exciting’ indicated their high expectations of the programme. As one of the members expressed it: ‘My hope is that it will generate a ripple effect that shows that this co‐created research was a good thing and that more patients can become involved in research… without having to be a researcher to begin with’ (I5, Round 3). This quote reflects a strong commitment to contribute to this social movement that was shared by programme members and suggests a hope that the programme might pave the way for further collaborations and patient‐led initiatives.

##### Challenging and changing the status quo

For some members, an important driving force for engaging in the research programme was the opportunity to challenge and change the traditional roles of patients and caregivers in healthcare and research. These members suggested that the programme could lead to a new way of conducting research, where individuals with first‐hand experience of the phenomenon of interest are collaborators, rather than ‘research subjects’. They expressed a hope that such research would become more relevant to society, and hopefully also influence clinical practice: ‘I hope we can learn something so powerful and unique that our learnings within the programme can actually have an impact on… on healthcare… with a clear starting point being patients or their informal caregivers' needs and situations and ideas’ (R5, Round 2). However, besides high expectations, there was initially also some skepticism towards the attitudes of the researchers in the programme; the fear was that the researchers may be primarily interested in advancing their own careers. The following quote illustrates how members engaged in reflections about these issues early in the research programme: ‘The overall purpose is to create something that is better for patients and caregivers. The main purpose is actually not to get publications. This is something we need to remind ourselves of, we who are more traditional researchers’ (R2, Round 1). Members experienced that the research programme provided an opportunity to change the traditional roles and practices for patients, caregivers, as well as researchers.

##### Facilitating knowledge creation and dissemination

The members predicted that the programme would contribute to learning on an individual level as well as within academia and society at large. As one member put it: ‘Researchers who participate will learn things that they thought they knew… or learn things that they were not aware that they didn't know about the patient perspective. I think that will be the greatest effect’ (I2, Round 2). This type of individual learning about the value of collaborators without formal research education was manifested 2 years into the programme:

*I've gotten a better understanding… that a researcher actually has reason to have great respect for the amateurs. I just use that word in a somewhat provocative sense. I mean, people who do not have many years of research training, but who I see as very, very valuable collaborators. As I described, I see that it enriches the research process*. (R2, Round 3)

Members also emphasized the importance of generating and disseminating needs‐based research findings that are relevant to the larger population that healthcare serves. They hoped that the programme would contribute to useful and applicable knowledge about how cocreation between patient innovators and researchers can be achieved in future initiatives.

#### 3.2.2 Going on a road trip together

##### Finding, redefining, and balancing roles

Questions of power and roles were raised in all interview rounds. Members experienced that the partnership approach implied that roles and responsibilities were not as clear‐cut as in traditional research with a top‐down leadership. In particular, the role of patient innovators in comparison to that of researchers was initially perceived as unclear:

*It is a bit different from a usual research project where you have [set roles] like researcher and study subject. Here we have the patient in the driver's seat… yes, but what does that entail for the one with the researcher's hat? What does it really entail, does the driver's seat apply to the innovation or to the entire project?* (R8, Round 1)

To find their roles in the team and figure out how to cocreate the programme, members experienced that they needed to step out of their comfort zones, which required an investment of time and effort. By the end of the first year, many described their roles as still developing and that the roles, in general, were dynamic. The members thought that the effort that was put into creating role definitions was helpful in providing clarity and direction, as well as managing different levels of involvement. By the end of the second year, the roles had become clearer or had changed in nature (e.g., by assuming new responsibilities). The patient innovator role may have undergone the greatest change as patient innovators were described as increasingly active in research and meetings, despite differences in preconditions:

*I can invest 10% of my time and you can invest 100% of your time; of course, different things will be expected of us and I will not be able to deliver in the same way. But this does not necessarily mean that we need to cocreate less. We just need to plan accordingly*. (I5, Round 3)

##### Acknowledging and respecting each other's baggage

The topics addressed in this programme carried more emotional weight than one generally attributes to work. This highlights the importance of acknowledging and respecting each other's baggage, which was explicitly done during the baggage claim exercise almost 1 year into the programme. Exploring each other's baggage revealed that several participants had prior experiences of patient involvement as a means of box‐ticking or being an ‘alibi’ for academia. This could explain perceptions that patient innovators were sometimes defensive over their innovations and reluctant to letting researchers in:

*The innovators have said it themselves that there is a lot of frustration… that their entry point is often frustration, and ours is quite neutral. We don't have baggage in the same way. So, I think it has been difficult to know how I as a researcher should deal with the emotional reactions that I am definitely not used to… or to a very limited extent. And I think that this has probably been the most challenging*. (R4, Round 2)

In addition to having more emotional baggage, members experienced that patient innovators had an emotional investment that was not always shared by the researchers: ‘for us patient innovators, our innovations are a bigger part of life than they are for the researchers’ (I2, Round 2). Despite openly communicating about experiences and expectations in the baggage claim exercise, the fear of traditional hierarchical roles was still a concern after the first programme year, potentially imposing a barrier to trust and cocreation among programme members. By the end of the second year, participants expressed less worry and emphasized that continuing to share and respect each other's experiences and concerns proved essential.

##### Driving as a team

The programme members gradually learned to drive as a team by sharing and listening to each other's perspectives, as well as engaging in continuous negotiations to gain common ground. Initially, members experienced confusion and disagreements regarding their mutual participation, and how to divide or share responsibility and make decisions. While some members felt that it was necessary to cocreate all parts of the programme, others explained that the most important things were transparency and the opportunity to participate on equitable terms when desired. There was agreement that everyone cannot be equally active, but members also felt that this should not hinder cocreation and the sharing of power. Learning to work as a team involved discovering how to leverage differences and balance expectations in a constructive way.

*To be open both about the opportunities and challenges we experience, and dare to discuss, dare to have conflicts sometimes or think differently… and see if we can meet by exploring each other's differences. We need each other somehow. No one can do everything on their own. We need a combination of experiences, knowledge, and competence. These things are found in the individuals somehow*. (R8, Round 1)

Patient innovators described that they learned about research processes and researchers gained an increased understanding of patients' perspectives on issues that matter to them. By the end of the second year, members expressed a shift in mindset as they started to view each other as individuals with different expertise, rather than two homogeneous groups consisting of researchers and patient innovators, respectively. One of the patient innovators highlighted this by emphasizing the need to walk in ‘everyone's shoes’. Although a feeling of co‐ownership and codriving of the research process emerged over time, a member stated that ‘it is still a challenge to walk a mile in another person's shoes… I think this will remain. And it should remain that way, because that's what it's all about’ (I2, round 3).

#### 3.2.3 Finding the tempo: from talking to doing

##### Concerns about reaching targets

The collective experiences of programme members illustrate that partnership research involves carefully balancing direction, productivity, and consensus building. Maintaining democratic processes in cocreation while also moving forward was repeatedly raised as a challenging endeavour, which also led to some worry over not achieving targets: ‘My feeling is that it's maybe been a bit too much that everyone should be involved in sharing their opinions, so we don't really get anywhere’ (I2, Round 2). Apart from democratic processes taking time, deviations in research focus could also be a cause of concern:

*This is about innovations that are crucial for patients and their caregivers… that have been developed and are now being implemented in healthcare. If we lose that focus, it would probably be because the researchers want to work on something theoretical that might become an article that never makes an impact*. (I6, Round 2)

The above quote again illustrates a concern that researchers would prioritize their academic careers above achieving the aims of the research programme.

##### Overcoming challenges

Cocreation of the partnership research programme was an explorative learning process that involved recognizing and managing conflicts and tensions that arose. Most conflicts arose at the beginning when expectations differed—especially between the patient innovators and researchers. One example was an application for ethical approval of the research, where the patient innovators were initially not involved in writing the application nor listed as participating researchers. The omission of patient innovators in this process was seen as a red flag by some, whereas others had viewed this as merely an administrative activity that did not require cocreation. The different views and expectations regarding ethical application contributed to early experiences of power imbalance and insufficient cocreation; the interviews also reveal feelings of insecurity or dissatisfaction among some members during the first programme year. The occurrence of challenges, and sometimes conflicts, was not surprising to the programme members: ‘It's not a question of whether we'll run into challenges, but rather it's about how we handle those that pop up. And I think that they, so far, they have really been handled very respectfully’ (I2, Round 3). Thus, conflict resolution was accepted as an integral part of cocreation. As highlighted in the following quote, challenges should be managed quickly and treated as opportunities for continuous learning:

*…it's like you have a match and it can start burning rather quickly. These kinds of things can happen to us, so it's important to manage these situations quickly so they don't grow into something really big. So in some ways we have learned a lot, but I don't think that this is something we will ever have finished learning… it continues to develop all the time because it is very, very complex and difficult*. (R4, Round 2)

##### Supporting structures for taking on speed

The programme had a long start‐up phase with a lot of time devoted to team building and developing organizational structures and routines. The recruitment of postdocs during the first term of the programme was identified as central to mobilizing these processes. Several members reported that given the dynamic and complex nature of the programme, clear routines and structure was needed, more than they had experienced in previous projects:

*We have quite a lot of structure, I would say. More than, what should I say, any regular research project… we didn't have any structure when we started and then the need to have certain structures emerged. And I think particularly from the perspective of innovators, who are not in research environments on a daily basis. They have primarily wanted to know things like: Where are decisions taken? Who is allowed to influence? Which mandates do we have? These kinds of questions are things they have wanted to know. And then we have together created these structures*. (R4, Round 3)

As illustrated in the above quote, the supporting structures that were important to programme members concerned mainly strategic questions, such as decision‐making and the ability to influence. The establishment of clear routines regarding these questions contributed to psychological safety, trust, and equity. In addition to structures and routines, a salary to compensate for everyone's time was considered particularly important for being able to collaborate on equitable terms. By the end of the second year, a member concluded that ‘it took pretty long to get started with all the projects in the programme. There was a lot in the beginning about creating routines… but now we're on our way’ (R11, Round 3).

## 4 DISCUSSION

This study described key developments, as well as the hopes, expectations, and experiences of patient innovators and researchers who jointly embarked on a 6‐year partnership research programme focusing on patient‐driven innovations. Following the use of a *driving* metaphor in the programme title (i.e., patients in the driver's seat), we continued to use this metaphor in the analysis. Our findings illustrate both the high hopes and expectations of programme members and the challenges that had to be managed when conducting partnership research in a group consisting of members with diverse backgrounds, experiences, and expertise. After 2 years of the partnership research, programme members' experiences may be summarized as *a rocky road but worth the drive*. At the time of writing this manuscript, we are more than 3 years into the partnership programme and have interpreted our findings based on the experiences we have gathered to this point, as well as related literature.

### 4.1 Expectations beyond research production

Programme members had high expectations that went beyond what is often expected from a research project or programme (e.g., scientific publications). The programme was viewed as an opportunity for individuals, as well as organizational and societal learning about how to practice partnership research. Members expressed hopes that the programme would contribute to changes in healthcare and society at large, towards a culture of more respect for experience‐based knowledge, thus changing the roles of patients and caregivers. By setting an example, members hoped that the programme would contribute to *paving the path to a better tomorrow*. The high expectations for programme outcomes can partly be explained by the partnership design—all members perceived that they had a real opportunity to make an impact. The programme title and the fact that the innovations were driven by patients shifted the power balance more to the patient innovators' side already from the start. The strong funding prospects (i.e., the amount and duration of funding) also provided excellent conditions for making an impact. In addition, the trends in society, healthcare, and funding of research in Sweden were highlighting the importance of involving patients and the public in research.^45^ A recent review confirms this trend by showing that apart from the United Kingdom, where PPI is most established in Europe, significant work is taking place in the Netherlands and Scandinavian countries.^46^ Thus, multiple forces were supporting this partnership research approach, which gave the members a feeling that a paradigm shift is possible, acknowledging the involvement of patients at the highest level of decision‐making, as ‘patient designers’.^3^ Jordan et al.^47^ emphasize the importance of building on expectations as a starting point for discussing roles and responsibilities in the team, as the failure to fulfil expectations could lead to disillusionment and disengagement. These expectations should also guide the evaluation of the impact beyond research productivity. Many evaluation tools for PPI in research and health system decision‐making have been published, mainly focusing on the evaluation of engagement processes and self‐reported impact.^48^

### 4.2 Central partnership practices

We labelled the programme as a partnership programme in the funding application, but at that stage, we had not designed any practices to enable cocreation. The analysis of our meeting protocols gives an understanding of how the partnership practices evolved. Three practices seemed central in implementing the principle of working in equitable partnerships.^20^ First, the programme management team constellation, consisting of equal numbers of patient innovators and researchers, was established as a response to the incident early in the programme when patient innovators were not involved in the ethical application process. By delegating future programme‐level decisions to the management team, we sought to create opportunities for more equitable power sharing in programme‐level decisions. Second, the establishment of specific task forces (e.g., the role description task force) involving patient innovators and researchers ensured the involvement of both perspectives in the programme administrative work. Third, making the research meetings open to all members in the programme enabled everybody to participate in the practical research work. What is central to all these practices is the aim to develop structures for supporting the sharing of power at different levels (i.e., when making programme‐level decisions, shaping the collaborative work environment, and performing research activities). Yet, we acknowledge that developing enabling structures is not sufficient to ensure power balance. Therefore, we also had in‐depth discussions about our various roles and responsibilities. The importance of clear definitions and understanding about roles is well documented.^49^ In our programme, the role description document evolved to a key document that we reviewed and updated regularly, in line with practice recommendations.^50^ Later in the programme, we have developed additional practices to support equitable distribution of power in the operative research work—from project proposal to write‐up. However, we acknowledge that our programme members also shared perceptions of having too many routines. Thus, a balance between providing structure and allowing creativity seems central. We believe that although other researchers may adopt and adapt the practices that we have developed, the process of mutually agreeing on practices was central for us, as also highlighted by Greenhalgh et al.^17^

### 4.3 Creating a supportive environment

The partnership practices implied changes in the traditional roles of researchers and patient innovators, which posed high demands on the members during the first 2 years. Patient innovators as well as researchers were reimbursed for their invested time, which has been described as a key aspect of organizational support for PPI.^49^ Although essential, financial security was not sufficient for creating a supportive environment. Navigating in the new territory led to feelings of uncertainty about what to expect from each other, and thus a general lack of clarity and control. Our analysis of meeting topics presented in Figure 1 illustrates how the *forming* and *storming* of roles occupied early discussions and settled in later stages.^51^ While trying to find their roles, programme members experienced tensions, misunderstanding, and even conflicts in the group. According to Williams et al.,^28^ ‘difficult conversations or disagreements can be a welcome sign that different views, values, perspectives and experiences are being considered and discussed as part of a relational process’ (p. 5). On the other hand, the experienced fear of tokenism, which remained throughout the study period, suggests that we struggled in our endeavour to support the egalitarian principle that is central to coproduction.^28^ A possible turning point in our group development was the baggage claim exercise, in which we openly shared prior experiences and current concerns, which members considered central in learning to understand each other's perspectives. By acknowledging and respecting each other's baggage, it was easier for us to establish trust and create a supportive environment conducive to collaboration. At the end of the second year, we observed a new perspective about the partnership programme, which may be a result of a more trusting environment. Members started to view the partnership as a collaboration between *individuals* with different experiences and expertise, which contrasts from our initial characterization of the research partnership as a collaboration between distinct *groups* (i.e., patient innovators and researchers). A possible explanation for this is that once members trusted that their expert roles were being acknowledged and respected, there was room for seeing behind these roles, focusing on other qualities. By shifting the focus from expert roles to individuals with unique experiences and expertise, we could argue that our findings may be relevant for any type of partnership research projects and not just those involving partnerships between researchers, patients, and caregivers.

### 4.4 Strengths and limitations

A strength of this study is the longitudinal design, which allowed us to capture how partnership practices and experiences developed over time. The involvement of authors as study participants may be viewed both as a strength and a limitation. As authors were involved in the interpretation of findings as ‘native researchers’,^52^ we had to carefully balance the benefit of authenticity against the risk of insufficient detachment from the data. Self‐reflection was supported by ensuring that both the patient innovator and researcher perspectives were represented when findings were discussed and reported. However, the patient innovator perspective was not represented in the initial design of the interview guide, which we acknowledge is a limitation of this study. For subsequent interview rounds, programme members (researchers and patient innovators) have been invited to contribute with adjustments to the interview guide. The importance of clarifying patient and public contributions in the research process, and as coauthors, has been emphasized in the community of patient advocates.^53^, ^54^ To assess the transferability of our findings to other settings, careful consideration of the context is required. In this article, we have used the term partnership to illustrate our ambition to create a research programme where researchers, patients, and caregivers together share power and responsibility for the research throughout, as proposed by Hickey et al.^21^ In this research programme, a partnership approach seemed like the only way forward as we focused on innovations initiated and driven by patients and caregivers. Thus, when interpreting the results, one must keep in mind that whether partnership research is a suitable solution for all research is not investigated in this study. Further, how roles and responsibilities are best distributed in a partnership programme may also differ depending on the setting. It should be considered that patient innovators are generally quite experienced with the healthcare system and actively engaged in improving self‐care and healthcare services for themselves and others, which are qualities shared by so‐called lead patients or second‐generation e‐patients.^55^, ^56^ Whereas the patient innovators in our programme desired strong influence on decision‐making in the programme, PPI research in other settings shows that this may not always be the case,^57^ highlighting the importance of adjusting partnership practices to the expectations of partnership members. More studies are needed that elucidate when (i.e., in what type of research study and when in a research process) partnership between researchers, patients, and caregivers is purposeful; how partnership is understood (i.e., what classifies or does not classify as partnership) by different actors; and the impact of partnership research beyond research productivity. It should also be acknowledged that we in this study did not explore the mechanisms for power sharing in detail. This could be further investigated by applying tools for evaluating group dynamics in partnership research, which includes the assessment of shared leadership, communication, mechanisms for recognizing and constructively resolving conflicts, and levels of trust and cohesion.^58^

## 5 CONCLUSIONS

Our longitudinal qualitative study, exploring key developments in a partnership research programme and the hopes, expectations, and experiences from patient innovators and traditional researchers during a 2‐year period, found that it was a rocky road, but worth the drive. Our findings emphasize the value of sharing, respecting, and acknowledging each other's experiences and concerns to build mutual trust and shape partnership practices together. The high expectations beyond research productivity suggest that we need to consider outcomes at different levels, from the individual to society, when evaluating the impact of partnership research.

## AUTHOR CONTRIBUTIONS

Carolina Wannheden, Carl Savage, and Henna Hasson conceptualized the study and all authors contributed to its design and methodology. Henna Hasson, Terese Stenfors, and Carl Savage drafted the interview guides. Jamie L. Luckhaus, Sara Riggare, and Carolina Wannheden analyzed the interview data. Hanna Jansson and My Sjunnestrand analyzed the documents. All authors contributed to interpreting the results. Jamie L. Luckhaus and Carolina Wannheden created visualizations. Henna Hasson, Jamie L. Luckhaus, Carolina Wannheden, Carl Savage, My Sjunnestrand, and Hanna Jansson drafted the first version of the manuscript. All authors commented on subsequent versions and read and approved the final manuscript.

## CONFLICT OF INTEREST STATEMENT

All authors, except Jamie L. Luckhaus, are members of the research programme that was studied.

## ETHICS STATEMENT

Ethical approval for the study was obtained from the Swedish Ethical Review Authority (reg nr. 2019‐03849).

## Data Availability

The data that support the findings of this study are available on request from the corresponding author. The data are not publicly available due to privacy or ethical restrictions.
